# Functional characterization of GplA (MSMEG-0394) reveals a novel determinant of glycopeptidolipids homeostasis in *Mycobacterium smegmatis*

**DOI:** 10.3389/fmicb.2026.1929615

**Published:** 2026-09-03

**Authors:** Milena Della Gala, Rosangela Marasco, Ida De Chiara, Martina Montebuglio, Luigi Russo, Severina Pacifico, Lidia Muscariello

**Affiliations:** 1 Department of Environmental, Biological, and Pharmaceutical Sciences and Technologies (DiSTABiF), University of Campania Luigi Vanvitelli, Caserta, Italy; 2 Institute of Biostructure and Bioimaging (IBB), National Research Council (CNR), Napoli, Italy

**Keywords:** cell-envelope homeostasis, glycopeptidolipids (GPL), GplA (MSMEG-0394), *Mycobacterium smegmatis*, non-tuberculous mycobacteria

## Abstract

**Introduction:**

Glycopeptidolipids (GPLs) are major constituents of the cell envelope of non-tuberculous mycobacteria (NTM) and play essential roles in colony morphology, surface motility, biofilm formation, antimicrobial susceptibility, and host–pathogen interactions. Although the GPL biosynthetic locus has been extensively characterized, the function of several conserved genes within this cluster remains unresolved. Here, we functionally characterized GplA (MSMEG-0394), a conserved small protein encoded within the GPL biosynthetic locus of *Mycobacterium smegmatis*.

**Methods:**

The role of GplA was investigated by combining genetic and phenotypic analyses, lipid profiling by thin-layer chromatography and electrospray ionization mass spectrometry, transcriptional analyses, complementation studies, and structural modelling.

**Results:**

Deletion of gplA resulted in extensive alterations of cell-envelope-associated phenotypes, including rough colony morphology, impaired sliding motility, delayed biofilm formation, increased cell aggregation, reduced growth, and enhanced susceptibility to rifampicin. Biochemical analyses demonstrated a marked remodeling of the GPL profile in the mutant strain, supporting a role for GplA in maintaining GPL homeostasis. Transcriptional analyses further showed that *gplA* is co-transcribed with *MSMEG_0393* and *MSMEG_0395* and that deletion of *gplA* results in upregulation of this operon, suggesting that GplA contributes, directly or indirectly, to the control of GPL-associated gene expression. Complementation substantially restored the wild-type phenotype, confirming that the observed alterations were specifically associated with loss of *gplA*. Finally, structural modelling predicts that GplA is a small soluble protein lacking recognizable catalytic features, consistent with a non-enzymatic role and supporting the hypothesis that it may act through protein-protein interactions.

**Discussion:**

Collectively, our findings identify GplA as a determinant of glycopeptidolipid homeostasis and suggest that this conserved small protein contributes to the coordination of cell-envelope composition, surface-associated physiology, and operon expression in *M. smegmatis*. These findings expand the current understanding of the regulatory network underlying GPL homeostasis and provide a framework for future studies aimed at defining the molecular mechanism of GplA function in clinically relevant NTM.

## 1 Introduction

The mycobacterial cell envelope is a highly dynamic multilayered structure that not only protects the bacterium from environmental stresses and antimicrobial agents but also governs surface-associated behaviours and interactions with the host. Its outer layer, enriched in glycolipids, contributes to the unique surface properties of different mycobacterial species. Among these glycolipids, glycopeptidolipids (GPLs) are particularly important and are produced by several NTM, including members of the *Mycobacterium avium* complex (MAC), *Mycobacterium abscessus*, *Mycobacterium chelonae*, *M. smegmatis*, *Mycobacterium fortuitum*, *Mycobacterium porcinum*, *Mycobacterium. senegalense*, *Mycobacterium xenopi*, and others (Gutiérrez et al., 2018; Pang et al., 2013). This intricate cell wall architecture plays a key role in drug resistance and pathogenicity, facilitating survival within host macrophage phagolysosomes, promoting antibiotic resistance, and enabling biofilm formation (Tursun et al., 2025). Extensive genetic and biochemical studies have defined the organization of the GPL biosynthetic locus in *M. smegmatis*, where nearly 30 genes are clustered within a 65-kb genomic region (Jeevarajah et al., 2002; Patterson et al., 2000; Tatham et al., 2012). These genes, many in operons, are responsible for the synthesis of the peptide core and fatty acid acyl chain, the modification of GPLs core (including glycosylation, methylation, acetylation), the assembly of various biosynthetic enzymes on the cell membrane, and the export of GPLs. GPL locus is conserved among NTM. Indeed, *M. abscessus*, *M. chelonae,* and MAC serovars have the GPL locus similar to that of *M. smegmatis*, although the genes involved in the synthesis of fatty acid chains are comparatively scattered (Gutiérrez et al., 2018; Johansen et al., 2020b; Pang et al., 2013). The high percentage of identity in the GPL gene cluster between other NTM and *M. smegmatis* makes the latter a useful model to study these compounds and to elucidate their relevance in mycobacteria of clinical significance. In recent decades, numerous experimental studies focusing on the phenotypic analysis of strains defective in GPL production have demonstrated their roles in many aspects of mycobacterial biology, including host-pathogen interaction (Roux et al., 2016; Shimada et al., 2006; Sweet et al., 2010; Whang et al., 2017), sliding motility (Etienne et al., 2002; Recht et al., 2000) and biofilm formation (Kocíncová et al., 2008; Recht et al., 2000; Tatham et al., 2012; Yang et al., 2017). The most commonly observed phenotypes in mutant strains defective in GPL production are the altered colony morphology, changes in cell wall hydrophobicity, and bacterial aggregation. These alterations of surface properties affect the motility, antibiotic susceptibility, and interactions with host cells. It is well known that members of NTM typically exhibit different colony morphologies, such as smooth (S) or rough (R), which are correlated to the virulence of the pathogenic strains. The S and R variants interact differently with host cells and exhibit different intracellular behaviours, as reported in human monocytes and other phagocytes (Howard et al., 2006; Roux et al., 2016). The loss of GPLs in R strains exposes immunostimulatory molecules, such as PIM2 and lipoproteins, triggering TLR-2-mediated inflammation and TNF expression, which can cause tissue damage. In contrast, S strains retain GPLs that mask these components, dampening early immune activation and promoting initial colonization. Moreover, *M. abscessus* R was found to be more apoptotic than *M. abscessus* S in different types of macrophages. The lack of GPLs leads to increased apoptosis, which promotes extracellular replication, acute infection, and the most severe forms of the disease. This also has detrimental consequences for the host since, by unmasking other pro-inflammatory cell-surface components, the loss of GPLs translates to severe inflammation and lung damage. Although GPLs are now widely recognized as common components in mycobacteria and play important roles in MAC and other clinically relevant species, their biosynthetic pathway is still not fully elucidated (Ryan and Byrd, 2018). Many reviews describe comparative genomic analysis of the GPL biosynthesis locus in NTM, particularly *M. abscessus*, *M. avium*, and *M. smegmatis* (Gutiérrez et al., 2018; Kim et al., 2013; Pang et al., 2013). Despite extensive characterization of the GPL biosynthetic pathway, several conserved genes within the GPL locus remain functionally uncharacterized. This gap limits our understanding of the regulatory network coordinating GPL homeostasis and, consequently, cell-envelope architecture and surface-associated physiology in NTM. Some genes of the GPL cluster are still uncharacterized and are reported in genome annotation as “unknown” or “hypothetical protein.” Here, we functionally characterize GplA (MSMEG-0394), a previously uncharacterized conserved protein encoded within the GPL biosynthetic locus of *M. smegmatis*. By combining genetics, phenotypic analyses, lipid profiling, transcriptional assays and structural modelling, we show that GplA contributes to GPL homeostasis and influences cell-envelope-associated physiology. Our findings identify GplA as a previously unrecognized contributor of GPL homeostasis and provide new insights into the regulatory network underlying cell-envelope adaptation in NTM.

## 2 Materials and methods

### 2.1 Bacterial strains and culture conditions

*Escherichia coli* TOP10 and DH5α were used in cloning experiments. *M. smegmatis* mc^2^155 was used throughout this work. *E. coli* strains were grown in standard Luria-Bertani (LB) broth, while the *M. smegmatis* strains were cultured in Middlebrook 7H9 broth (MB7H9-DIFCO), supplemented with 10% albumin-dextrose-catalase (ADC-BECTON DICKINSON), 0.2% glycerol (v/v), and 0.05% Tween 80, or in TY medium (8 g/L NaCl, 10 g/L tryptone, 5 g/L yeast extract, and 0.05% Tween 80). Additional growth media used for mycobacterial strains included tryptic soy broth (TSB-OXOID) and Sauton’s medium (0.5 g KH_2_PO4, 0.5 g MgSO_4_·7H_2_O, 2.0 g citric acid, 0.05 g ferric ammonium citrate, 60 mL glycerol, 4.0 g asparagine, 0.1 mL ZnSO_4_ 1% solution, final pH 7.4) which were used for the evaluation of surface-associated phenotypes. All strains were cultured at 37 °C with shaking at 200 rpm. When required, selective agents were added as follows: hygromycin B (200 μg mL^−1^ for *E. coli* and 100 μg mL^−1^ for *M. smegmatis*), kanamycin (50 μg mL^−1^ for *E. coli* and 25 μg mL^−1^ for *M. smegmatis*), 5-bromo-4-chloro-3-indolyl-*β*-d-galactopyranoside (X-gal 50 μg mL^−1^), and sucrose (2% w/v). For MIC determination, antibiotic stock solutions were prepared as follows: rifampicin and chloramphenicol (10 mg mL^−1^) in methanol; isoniazid, ethambutol, and streptomycin (10 mg mL^−1^) in filter-sterilized distilled water; ciprofloxacin and norfloxacin (10 mg mL^−1^) in 1 M NaOH.

### 2.2 3D structure prediction and dynamics studies of MSMEG-0394 and MAB-4102c by computational methodologies

The three-dimensional structure of MSMEG-0394 and its homolog MAB-4102c was predicted on the basis of the primary sequence using Alphafold v3.0 (Abramson et al., 2024) with default parameters. Thus, the calculation was performed using multiple sequence alignment (MSA) searching, unpaired mode for obtaining, for each protein, separate MSA and no filter options were applied for the minimum coverage with query and minimum sequence identity with query. For both proteins, the structural models, generated using default settings (i.e., number of models = 5; max_recycles = 3), were very similar and the one with the highest rank based on pLDDT (predicted Local Distance Difference Test) was considered as reference structural model and was used for further analysis. The identification of the secondary structure elements was performed using the software DSSP (Joosten et al., 2011). The reference 3D structural model obtained for each of the two proteins under investigation was evaluated and visualized using the following software: PROCHECK (Laskowski et al., 1996), PyMOL (Delano, 2002), and Chimera (Pettersen et al., 2004). The dynamical peculiarities of both proteins were investigated using Molecular Dynamics simulations techniques. In particular, simulations were performed by GROMACS (Hess et al., 2008) software using Amber99SB (Hornak et al., 2006) force field and explicit TIP3P water. For both MSMEG-0394 and MAB-4102c, 20 ns simulations were performed at 298 K. For each protein, the representative structural model predicted using the Alphafold protocol was used as starting point. The Coulombic electrostatic potential was calculated for MSMEG-0394 and MAB-4102c using the software Chimera (Pettersen et al., 2004).

### 2.3 Isolation of the *M. smegmatis ΔMSMEG_0394* mutant strain

Plasmids and primer sequences used throughout this work are listed in Tables 1 and 2, respectively. Unless otherwise specified, all primers were designed using the Eurofins Genomics online primer design tool (Eurofins Genomics, Ebersberg, Germany) and synthesized by Eurofins Genomics. The *M. smegmatis* (*ΔMSMEG_0394*) mutant strain, carrying an *in-frame* deletion within the *MSMEG_0394* coding sequence, was isolated using a two-step homologous recombination method as previously described (De Siena et al., 2020). The upstream (up) and downstream (dw) flanking regions of *MSMEG_0394* were amplified by PCR using 100 ng of genomic DNA extracted from *M. smegmatis* mc^2^155 (wt) as the template, 1 × Phusion Flash High-Fidelity PCR Master Mix (Thermo Scientific), and 10 μM of each primer in a final reaction volume of 25 μL. PCR amplifications were carried out under the cycling conditions described in Table 3. The 810 bp DNA fragment (up), corresponding to the upstream flanking regions of *MSMEG_0394,* was amplified using primers upMS0394f and upMS0394r, cloned into the *Hind*III and *Kpn*I restriction sites of the p2NIL vector, yielding the recombinant plasmid pMG01. Similarly, a 750 bp DNA fragment (dw), corresponding to the downstream flanking region of *MSMEG_0394,* was amplified using primers dwMS0394f and dwMS0394r, cloned into the *Kpn*I and *Pac*I restriction sites of pMG01, yielding plasmid pMG02. The counter-selectable marker cassette pGOAL19 was subsequently cloned into the *Pac*I site of pMG02 to generate the suicide delivery vector, pMG03. Plasmid pMG03 was introduced into *M. smegmatis* mc^2^155 wild-type via electroporation using a Bio-Rad Gene Pulser II electroporator (Bio-Rad, CA, United States) using the following parameters: 2.5 kV, 600,Ω and 25 μF. Transformants exhibiting a single-crossover (SCO) integration event were identified by their blue phenotype on TY agar plates supplemented with X-gal, and by resistance to both kanamycin and hygromycin. A single SCO-positive clone was cultured in TY antibiotic-free medium at 37 °C for 5 days to enable the second recombination event. The resulting colonies were selected on TY agar plates supplemented with sucrose and X-gal. White, sucrose-resistant colonies were screened for sensitivity to kanamycin and hygromycin and further verified by colony PCR to confirm excision of the internal segment of *MSMEG_0394*, resulting in a 228 bp *in-frame* deletion. A confirmed deletion mutant was selected for downstream analyses and is hereafter referred to as *M. smegmatis* (*ΔMSMEG_0394)*. The deletion was also verified by Sanger sequencing (Eurofins Genomics, Ebersberg, Germany).

**Table 1:** Plasmids used in this work.

Name	Applications	Description	Reference
p2NIL	*M. smegmatis ΔMSMEG_0394* isolation	A high copy suicide vector with Kanamycin resistance	(De Siena et al., 2020)
pMG01	*M. smegmatis ΔMSMEG_0394* isolation	p2NIL + *MSMEG_0394* upstream region	This work
pMG02	*M. smegmatis ΔMSMEG_0394* isolation	pMG01 + *MSMEG_0394* downstream region	This work
pGOAL19	*M. smegmatis ΔMSMEG_0394* isolation	Marker Cassette, conferring ampicillin resistance, sucrose sensitivity, and carrying the *LacZ* gene	(De Siena et al., 2020)
pMG03	*M. smegmatis ΔMSMEG_0394* isolation	pMG02 + pGOAL19 cassette	This work
pMV306hsp	*M. smegmatis ΔMSMEG_0394* complementation	Mycobacteria integrating vector with *hsp60* promoter	(De Siena et al., 2020)
pMG04	*M. smegmatis ΔMSMEG_0394* complementation	pMV306hsp + *MSMEG_0394* coding sequence	This work

**Table 2:** Oligonucleotides used in this work.

Name	Sequence	Description
Isolation of the *M. smegmatis ΔMSMEG_0394* mutant strain		
upMS0394f	5′-CCCAAGCTTTCTTCCTCAATTACGGGTACG −3′	Forward primer for the amplification of the upstream *MSMEG_0394* fragment
upMS0394r	5′-CGGGGTACCCAGGTTCATGCCCTCACTATA −3′	Reverse primer for the amplification of the upstream *MSMEG_0394* fragment
dwMS0394f	5′-CGGGGTACCGCCGTCGACGGAAAATAG-3′	Forward primer for the amplification of the downstream *MSMEG_0394* fragment
dwMS0394r	5′-CCTTAATTAAATGCAACATTTGCCGACG −3′	Reverse primer for the amplification of the downstream *MSMEG_0394* fragment
RT-PCR and RT-qPCR analyses		
Ctrldw0394f	5’-GACAAGAACTTCGACGCGG-3′	Forward primer for *MSMEG_0393/94/95* co-transcription analysis
Ctrlup0394r	5’-GCTGACATGATCAACCCG-3′	Reverse primer for *MSMEG_0393/94/95* co-transcription analysis
Ctrlup0394f	5’-CAAAGTCGCGCAACAAGC-3′	Forward primer for *MSMEG_0393* gene (qRT-PCR analysis)
Up0394r	5′-CAAAAGTTGATGCCGTCCGAG-3′	Reverse primer for *MSMEG_0393* gene (qRT-PCR analysis)
RTMS2758f	5’-CCAAGGGCTACAAGTTCTCG-3′	Forward primer for *MSMEG_2758* (*sigA*)
RTMS2758r	5’-CTTGTTGATCACCTCGACCA-3′	Reverse primer for *MSMEG_2758* (*sigA*)
Isolation of the *M smegmatis ΔMSMEG_0394* complemented strain		
cMS0394f	5′-CGGAATTCAGAAGGAGAAGTACCGATGAACCTGGTGCAG-3′	Forward primer for the amplification of *MSMEG_0394* gene
cMS0394r	5′-CCCAAGCTTCTATTTTCCGTCGACGGC-3′	Reverse primer for the amplification of the *MSMEG_0394* gene

**Table 3:** PCR conditions.

Primer	Melting	Annealing	Elongation	Cycles *n*°
Isolation of the *M. smegmatis ΔMSMEG_0394* mutant strain				
upMS0394f upMS0394r	98 °C, 1 s	70 °C, 5 s	72 °C, 40 s	30
dwMS0394f dwMS0394r	98 °C, 1 s	70 °C, 5 s	72 °C, 40 s	30
Isolation of the *M. smegmatis* complemented strain				
cMS0394f cMS0394r	98 °C, 1 s	70 °C, 5 s	72 °C, 40 s	30
RT-PCR analysis of the transcriptional unit in wild-type, mutant, and complemented strains				
Ctrldw0394f Ctrlup0394r	95°C, 15 s	56 °C, 15 s	72 °C, 1 min	35
Ctrlup0394f Up0394r	95°C, 15 s	56 °C, 15 s	72 °C, 1 min	35

### 2.4 Isolation of the *M. smegmatis* (*ΔMSMEG_0394_*pMG04) complemented strain

To restore *MSMEG_0394* expression in the *ΔMSMEG_0394* mutant, the full-length 255 bp coding sequence of *MSMEG_0394*, which includes both the start and stop codons, was amplified by PCR from *M. smegmatis* mc^2^155 genomic DNA using primers cMS0394f and cMS0394r (Tables 2 and 3). The forward primer was designed to include an optimized Shine-Dalgarno ribosome-binding site as previously described (De Siena et al., 2020). The resulting 283 bp fragment was subsequently cloned into the *EcoR*I and *Hind*III restriction sites of the integrative mycobacterial expression vector pMV306hsp (Addgene #26155), which drives gene expression under the control of the *Mycobacterium bovis* BCG *hsp60* promoter, yielding plasmid pMG04. The integrity of the recombinant construct pMG04 was verified by PCR and Sanger sequencing. The plasmid was introduced into *M. smegmatis ΔMSMEG_0394* by electroporation, generating the complemented strain *M. smegmatis ΔMSMEG_0394*_pMG04. Transformants were selected on kanamycin-containing medium, and the presence of the integrated plasmid was verified by PCR analyses using primers cMS0394f and cMS0394r (Tables 2 and 3).

### 2.5 RNA isolation and reverse transcription reactions

Total RNA for RT-PCR and RT-qPCR experiments was isolated from *M. smegmatis* mc^2^155 (wild-type), *M. smegmatis ΔMSMEG_0394*, and *M. smegmatis ΔMSMEG_0394*_pMG04 strains grown up to log-phase (OD_600_ 0.4) under standard growth conditions. Bacterial pellets were collected by centrifugation at 3273 rcf for 15 min at 4 °C, resuspended in 4 mL of RLT lysis buffer (Qiagen), and mechanically disrupted by vigorous vortexing in presence of glass beads. Total RNA was purified using the RNeasy columns (Qiagen) following the supplier’s instructions. To eliminate residual genomic DNA, RNA samples were treated with RNase-free DNase I (New England BioLabs) for 10 min at 37 °C, followed by phenol-chloroform extraction. The quality and quantity of RNA were evaluated using the NanoDrop spectrophotometer (Nanodrop, Thermo Scientific) and confirmed by agarose gel electrophoresis. Reverse transcription was performed using 2 μg of total RNA in a 40 μL reaction volume at 42 °C for 15 min, employing the QuantiTect Reverse Transcription kit (Qiagen). Samples without the reverse transcription reaction were used to confirm the absence of genomic DNA contamination.

### 2.6 RT-PCR and RT-qPCR analysis of the transcriptional unit in wild-type, mutant, and complemented strains

To investigate the transcriptional organization of the *MSMEG_0393/94/95* locus in *M. smegmatis* mc^2^155 (wild-type) we performed RT-PCR. In the mutant (*ΔMSMEG_0394*) and complemented strains (*ΔMSMEG_0394*_pMG04), RT-PCR analysis was used to verify that transcription of the flanking genes (*MSMEG_0393* and *MSMEG_0395*) was maintained, ensuring that neither the in-frame deletion nor the complementation disrupted the expression of adjacent loci. Primer pairs spanning the intergenic regions between the three genes were designed to detect co-transcribed mRNA species (Table 2), and reactions were carried out on cDNA synthesized from total RNA as previously described, with genomic DNA used as a positive control to confirm primer specificity. PCR amplifications were performed in a final reaction volume of 25 μL containing 2 μL of cDNA template (see 2.5 section), 1 × PCR buffer, 0.4 μM of each primer, and 0.5 U Taq™ Red DNA Polymerase (Bioline). Amplification was carried out under the cycling conditions described in Table 3. To quantify changes in operon expression, RT-qPCR was performed using a primer pair targeting the ORF of the first gene, *MSMEG_0393* (Table 2). Amplification was conducted with SYBR Green chemistry on a StepOnePlus RT-qPCR System (Applied Biosystems). The *sigA* gene was used as an internal control to normalize transcript levels. Thermal cycling conditions included: denaturation at 95 °C for 10 min, 40 amplification cycles at 95 °C for 15 s, annealing at 60 °C for 1 min, and elongation 30 s at 72 °C. All reactions were performed in triplicate to ensure reproducibility. Standard curves for both target and reference genes were generated by amplifying ten-fold serial dilutions of the cDNA templates. The *sigA* gene was used to normalize the relative expression level, calculated using the 2^-ΔΔCt^ method, as described by Livak and Schmittgen (2001).

### 2.7 Phenotypic characterization of *M. smegmatis ΔMSMEG_0394*

To investigate the physiological role of the *MSMEG_0394* gene, the *ΔMSMEG_0394* mutant strain was subjected to a series of phenotypic assays, including analyses of growth rate, colony morphology, biofilm formation, sliding motility, auto-aggregation, and surface-exposed lipid composition. The wild-type (*M. smegmatis* mc^2^155) and the complemented strain (*ΔMSMEG_0394_* pMG04) were included in all assays as controls to assess the phenotypic impact of the gene deletion and the extent of phenotypic restoration upon complementation.

#### 2.7.1 In vitro growth rate and colony morphology evaluation

For the growth rate analysis, mid-log phase cultures were inoculated into fresh TY medium supplemented with 0.05% Tween80 at a starting optical density (OD_600_) of 0.05. Kanamycin (25 μg mL^−1^) was added to the complemented strain. Cultures were incubated at 37 °C with shaking at 200 rpm, and growth was monitored by measuring OD_600_ at an interval of 3 h over a 45 h period using a UV-6300PC Double Beam Spectrophotometer (VWR, Milan, Italy). Colony morphology was evaluated using a Congo red plate assay, adapted with minor modifications from a previously described protocol (Deshayes et al., 2005). Briefly, mycobacterial cultures were grown up to the stationary phase, shortly vortexed with glass beads to enhance cellular homogeneity, and harvested by centrifugation at 3273 rcf for 10 min at 4 °C. The resulting cell pellets were washed once with 1X phosphate-buffered saline (PBS) to remove Tween80, and subsequently resuspended in the same buffer to a final OD_600_ of 1.0. Aliquots (5 μL) of the cell suspensions were spotted onto Congo red-supplemented TY agar (TY basal medium containing 1.5% agar, 0.02% glucose, 100 μg mL^−1^ ampicillin, and 100 μg mL^−1^ Congo red-sodium salt of 3,3′-([1,1′-biphenyl]-4,4′-diyl)bis(4-aminonaphthalene-1-sulfonic acid), supplied from Sigma-Aldrich. Plates were incubated at 37 °C for 5 days, after which colony morphology was visually assessed and compared across strains.

#### 2.7.2 Biofilm formation and sliding motility assay

Biofilm formation assay was conducted as previously described, with some modifications (De Siena et al., 2020). Wild-type, *ΔMSMEG_0394*, and the complemented strains (*ΔMSMEG_0394_*pMG04) of *M. smegmatis* were initially cultured in TY medium supplemented with 0.05% Tween 80 until reaching an optical density at 600 nm (OD_600_) of approximately 1.5. Cultures were then centrifuged at 3273 rcf for 15 min (room temperature) and washed twice with Sauton medium to remove residual Tween80. A dilution 1:500 of each culture was inoculated into 5 mL Sauton medium supplemented with 2% glycerol and dispensed into 60 mm diameter polystyrene Petri dishes. The plates were sealed with Parafilm and incubated at 37 °C in a humidified incubator for 7 days, then surface pellicle formation was qualitatively assessed and imaged. For the sliding motility assay, the protocol was adapted from previously reported methods (Wani et al., 2023). Briefly, 5 μL of the log-phase culture were spotted onto MB7H9 plates solidified with 0.8% ultrapure agarose (Bio-Rad) and supplemented with 2% glucose. Spreading was evaluated visually after incubation of parafilm-sealed plates at 37 °C in a humidified incubator for 1 week and imaged.

#### 2.7.3 Antibiotic susceptibility assay

Minimum inhibitory concentrations (MIC) for anti-TB drugs rifampicin, isoniazid, ethambutol, chloramphenicol, streptomycin, ciprofloxacin, and norfloxacin were determined with the REMA method previously described (De Siena et al., 2020). Briefly, 96-well flat-bottom microtiter plates were prepared by dispensing 50 μL of TY medium into each well, excluding those in the first column. Antibiotic stock solutions were prepared at double the desired final concentration, and 100 μL were added to the first column. A two-fold serial dilution was performed across the plate by transferring and mixing into wells containing only medium, up to the last second column. The last column served as an antibiotic-free growth control. *M. smegmatis* strains were grown in TY medium to mid-logarithmic phase (OD600 ≈ 0.6), diluted 1:1,000 in fresh medium, and 50 μL of the diluted culture were added to each well to achieve the final antibiotic concentrations. Plates were sealed with parafilm to prevent evaporation and incubated at 37 °C with gentle shaking (100 rpm) for 40 h. After incubation, 30 μL of filter-sterilized Resazurin sodium salt solution (final concentration: 0.2 mg mL^−1^) were added to each well. Plates were incubated for an additional 6 h under the same conditions, then imaged. MIC values were defined as the lowest antibiotic concentration at which no color change (from blue to pink) was observed, indicating inhibition of bacterial growth and metabolic activity.

#### 2.7.4 Autoaggregation assay

The autoaggregation assay was performed based on previously described methodologies, with slight modifications, to evaluate cell–cell interactions in the *M. smegmatis* strains (Deshayes et al., 2005). Cultures were grown to the stationary phase in tryptic soy broth (TSB) supplemented with 0.05% Tween 80, followed by centrifugation to separate single-cell suspensions from aggregated cells (10 rcf, 2 min at room temperature). The optical density at 600 nm (OD_600_) of the supernatant was measured and compared to the OD_600_ of resuspended samples in which aggregates were dispersed by vigorous vortexing with sterile glass beads. The aggregation index was calculated as the ratio between the two OD_600_ values (Deshayes et al., 2005). All assays were performed in triplicate.

#### 2.7.5 Lipid extraction and thin-layer chromatography (TLC) analysis

Glycopeptidolipids (GPLs) were extracted from *M. smegmatis* cultures according to previously described methods (Wani et al., 2023), with slight modifications. Briefly, bacterial cultures were grown to the stationary phase in 50 mL of TY basal medium supplemented with 0.05% Tween 80. Cells were harvested by centrifugation at 3273 rcf for 15 min at room temperature (RT) and washed with 10 mL of phosphate-buffered saline (PBS). GPLs were extracted by treating the cell pellets with a chloroform/methanol mixture (2:1, *v:v*) at 20 μL per mg of wet cell weight. The suspension was sonicated for 30 min (Branson Ultrasonics™ Bransonic™ M3800-E, Danbury, CT, United States) and incubated overnight at RT under agitation. After incubation, samples were centrifuged (3,273 rcf, 15 min, room temperature), and the organic phase containing GPLs was collected, dried using a rotary evaporator (Heidolph Hei-VAP Advantage, Schwabach, Germany), weighed, and resuspended in chloroform for subsequent analyses. An aliquot of 5 μL from each extract underwent thin-layer chromatography (TLC) on aluminium-backed silica gel 60 plates with fluorescent indicator UV_254_ (MERCK, Darmstadt, Germany). Plates were developed in chloroform/methanol (19:1, *v:v*), air-dried, and then sprayed with a solution of sulfuric acid:acetic acid:water (1:20:4, *v:v:v*). The plates were charred at 120 °C until characteristic coloration appeared, revealing the lipid profiles.

#### 2.7.6 Direct infusion electrospray ionization mass spectrometry (ESI-MS) analysis

Lipid extracts were analyzed by direct infusion electrospray ionization mass spectrometry (ESI-MS) using an AB Sciex Triple Quad™ 3,500 mass spectrometer equipped with an ESI source. Samples were diluted in acetonitrile containing 0.1% (*v/v*) formic acid and introduced into the mass spectrometer by direct infusion at a constant flow rate of 20 μL min^−1^ using a syringe pump.

Mass spectrometric analyses were performed in both negative- and positive-ion modes to allow complementary detection of GPL-associated species. In negative-ion mode (ESI-), the ion spray voltage (IS) was set to −4,500 V, while in positive-ion mode (ESI+) the ion spray voltage was set to +5,500 V. In both modes, the curtain gas (CUR) was set to 30, nebulizer gas (GS1) to 40, auxiliary gas (GS2) to 50, and the source temperature (TEM) to 500 °C. The declustering potential (DP) was set to −70 V in ESI- and +70 V in ESI+, while the entrance potential (EP) was maintained at −10 V and +10 V, respectively. Full-scan (Q1) mass spectra were acquired in profile mode over an *m/z* range of 950–1,800, with unit mass resolution, a scan rate of 2,000 Da s^−1^, and a step size of 0.10 Da. Data acquisition parameters were kept identical across strains and ionization modes to enable direct qualitative comparison of GPL profiles. Product ion (MS/MS) experiments were performed exclusively in positive-ion mode for selected precursor ions to evaluate their fragmentation behavior. Collision-induced dissociation (CID) was carried out in the collision cell using nitrogen as collision gas, with a collision energy (CE) set to 35 eV. All data were collected and processed using Analyst^®^ Software version 1.7.1 (AB Sciex).

### 2.8 Statistical analysis

The statistical analyses and graphs were prepared using Prism (8.0) (GraphPad Software). The bar plots in the graphs report mean values (±SD). The tests of significant difference were analyzed using One-way ANOVA with Sidak’s multiple comparisons.

## 3 Results

### 3.1 Conservation and divergence within the *MSMEG_0394* genomic region

**Figure 1:**
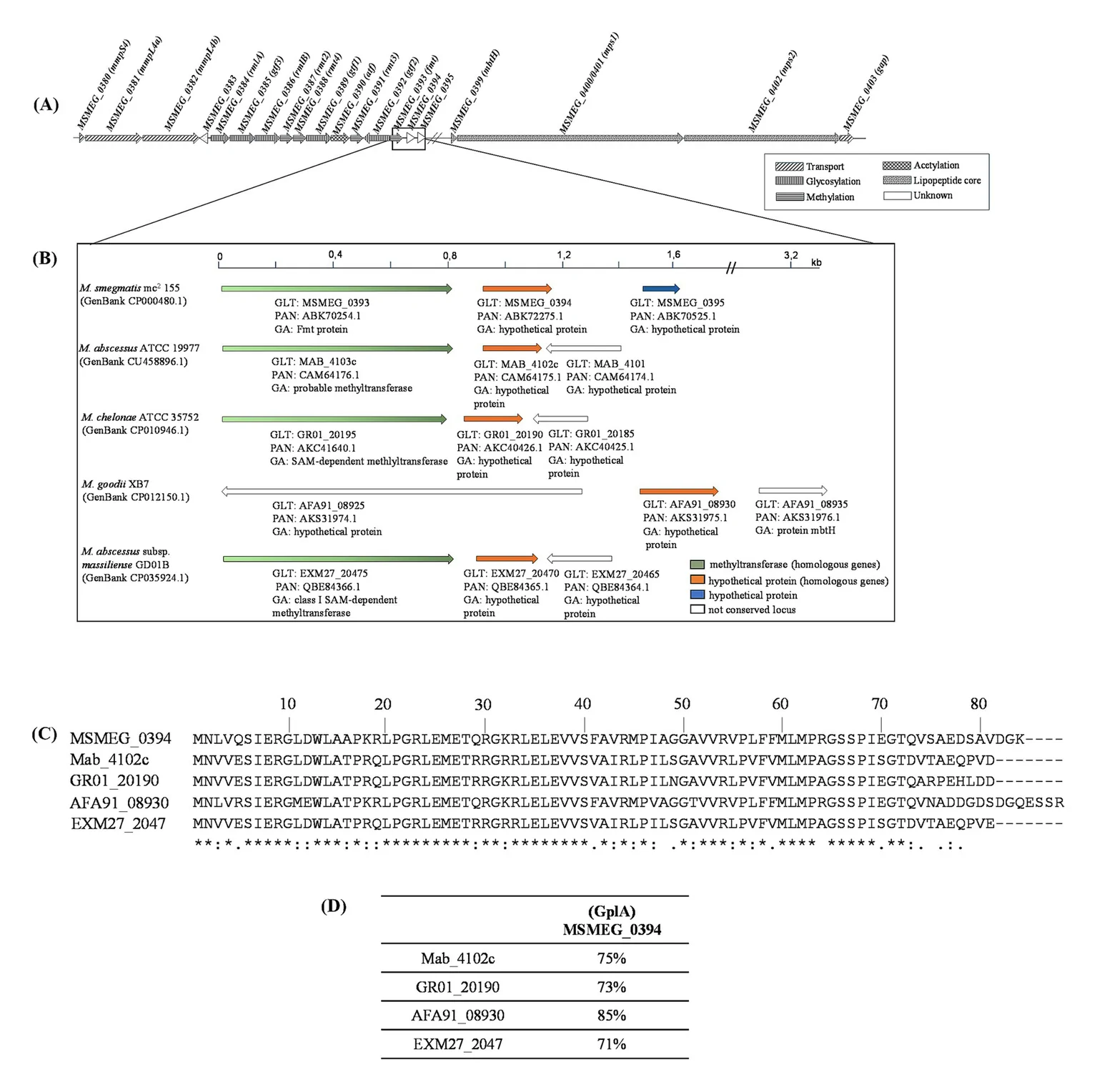
Comparative analysis of *MSMEG_0393/94/95* genomic locus in *Mycobacterium* spp. **(A)** Partial view of the 65 kb GPL genomic region in *M. smegmatis*, showing a 40 kb segment (from nucleotide 425,224 to 466,702) with 21 genes involved in GPL synthesis, modification, and transport (modified from Morgane Illouz et al. (2023)). **(B)** Comparative analysis of *MSMEG_0394* gene locus in *M. smegmatis* and its orthologs in *M. abscessus*, *M. chelonae*, *M. goodii*, and *M. abscessus* subsp. *massiliense*. Nucleotide sequence accession numbers (GenBank), protein accession numbers (PAN), available genomic locus tags (GLT), and genome annotation (GA) are reported. **(C)** Clustal W (Lasergene software, DNASTAR, Inc) alignment and **(D)** percentage identity of deduced amino acid sequence of MSMEG_0394 with MAB_4102c, GR01_20190, AFA91_08930, and EXM27_20470. Symbols: “*” Full conservation, “:” strong similarity, “.” weak similarity.

The glycopeptidolipids (GPLs) biosynthetic locus in *M. smegmatis* comprises a contiguous genomic region of approximately 65 kb, encoding nearly 30 genes, including multiple tailoring enzymes responsible for glycosylation, methylation, and acetylation. This compact genomic organization reflects the high structural complexity of GPLs and underscores the necessity for tight regulatory control to ensure the ordered progression of enzymatic steps during GPLs assembly (Ripoll et al., 2007). Despite detailed functional characterization of several biosynthetic enzymes (Illouz et al., 2023; Miyamoto et al., 2006; Tatham et al., 2012), a subset of genes within the GPL locus remains annotated as “hypothetical proteins” or of “unknown function.” Among these, particular attention was directed to the open reading frame *MSMEG_0394*, hereafter referred to as *gplA* (Figure 1A). Comparative genomic analyses revealed that the genomic region encompassing *gplA* is partially conserved across *M. abscessus*, *M. chelonae*, *M. goodii*, and *M. abscessus* subsp. *massiliense* (Figure 1B). Within these taxa, orthologous *gplA*-like open reading frames encoding peptides of 81–88 amino acids were identified, a putative protein size more consistent with a conserved structural or regulatory function rather than with catalytic activity. Multiple sequence alignments revealed a high degree of amino acid sequence identity (between 71 and 85%) of GplA and its orthologs (Figures 1C,D). Genomic context analysis further revealed that in *M. smegmatis, gplA* is located downstream of a gene encoding a fatty-acid O-methyltransferase (*MSMEG_0393*). This methyltransferase exhibits 74–85% sequence identity with the homologues in *M. abscessus*, *M. chelonae*, and *M. abscessus* subsp. *massiliense*, but is conspicuously absent from *M. goodii*. In contrast, *MSMEG_0395*, which lies immediately downstream of *gplA* and encodes an hypothetical protein, lacks orthologs in *these Mycobacteria* species. These observations suggest that *gplA* may represent a conserved, yet functionally uncharacterized element of the GPL locus, potentially acting as a structural or regulatory component. Its evolutionary conservation contrasts with the lineage-specific distribution of neighboring genes, suggesting that *gplA* performs a conserved function associated with GPL homeostasis, whereas the flanking genes might reflect adaptive diversification within different mycobacterial species.

### 3.2 *MSMEG_0394* is co-transcribed with the flanking genes *MSMEG_0393* and *MSMEG_0395*

**Figure 2:**
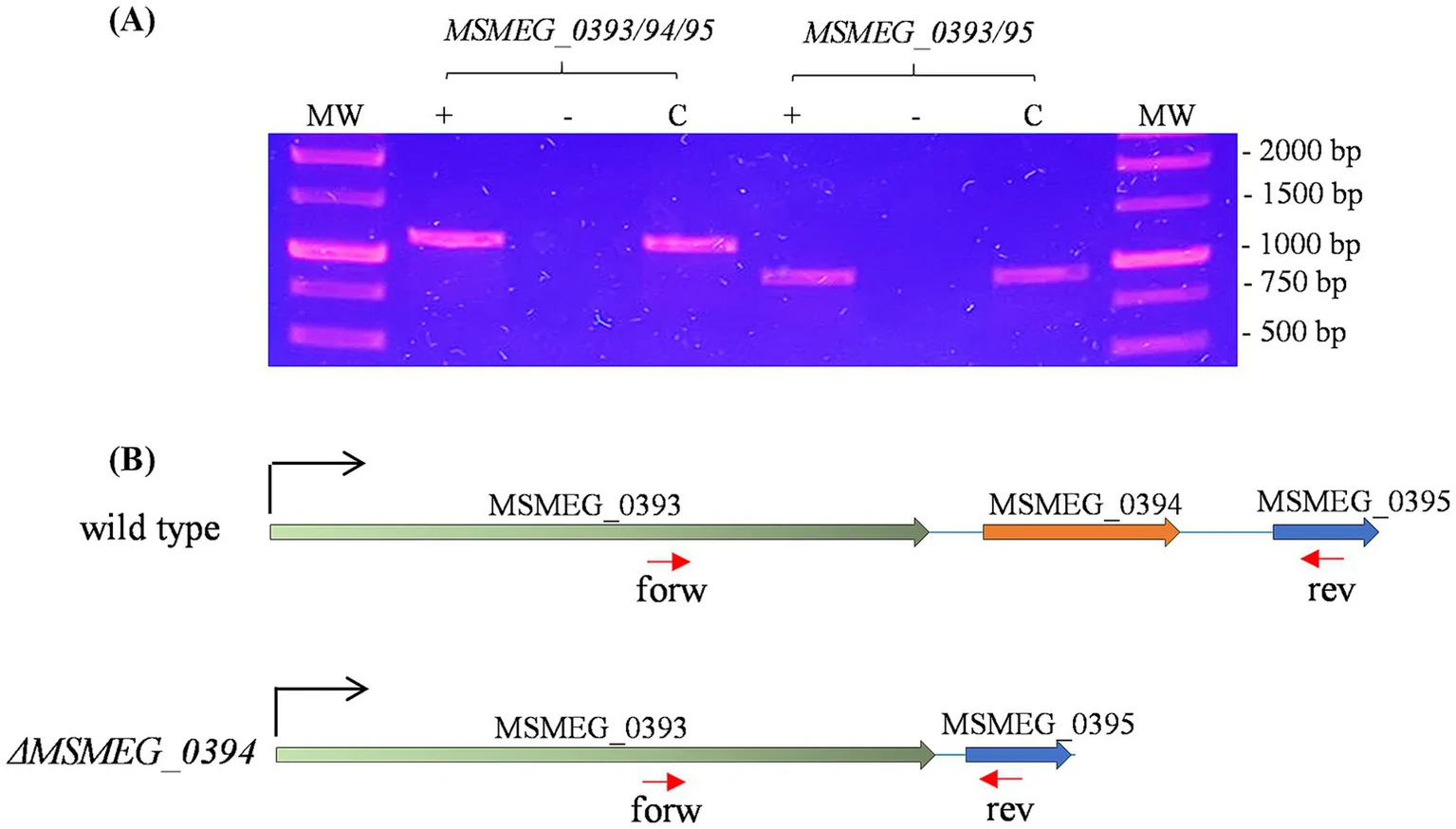
Transcriptional analysis of the *MSMEG_0393/94/95* operon. **(A)** RT-PCR experiments performed on total RNAs extracted from wild type (*MSMEG_0393/94/95*) and Δ*MSMEG_0394* (*MSMEG_0393/95*) strains. Each set of three reactions contains a positive control with genomic DNA as the template (C), a negative control without reverse transcriptase (−), and total RNAs with reverse transcription step (+). MW, molecular weight marker (1 kb Plus Opti-DNA Marker, abm). **(B)** Schematic representation of transcriptional units of the *M. smegmatis* wild type and Δ*MSMEG_0394* strains. Black arrows indicate direction of transcription. Red arrows indicate primer pairs (forw and rev) designed to detect co-transcribed genes, as reported in Table 2 of materials and methods section.

To elucidate the transcriptional organization of the *MSMEG_0394* locus, RT-PCR was conducted on total RNA extracted from log-phase *M. smegmatis* mc^2^155 using oligonucleotide pairs specifically designed to detect co-transcribed genes (Figure 2A and 2B; Supplementary Figure S1). Such operonic arrangements are a common feature of mycobacterial GPLs clusters, where structural and tailoring enzymes are co-expressed to coordinate complex modifications, such as glycosylation, methylation, and acetylation, thereby maintaining the structural diversity and functional integrity of GPLs (Deshayes et al., 2005; Ripoll et al., 2007). This organization minimizes the risk of imbalanced enzyme production, which could compromise critical cell envelope properties, including motility, biofilm formation, and environmental adaptability. Comparable operonic structures are observed in GPL clusters of other rapid-growing nontuberculous mycobacteria, such as *M. abscessus* and *M. chelonae*, underscoring the evolutionary conservation of coordinated gene expression in this pathway. Importantly, analysis of a *ΔMSMEG_0394* null mutant demonstrated that the upstream *MSMEG_0393* and downstream *MSMEG_0395* genes remain transcriptionally active, confirming that the deletion does not cause polar effects.

### 3.3 Deletion of the *gplA* gene alters *M. smegmatis* growth rate, colony morphology, sliding motility, biofilm formation, autoaggregation, and antibiotic susceptibility

**Figure 3:**
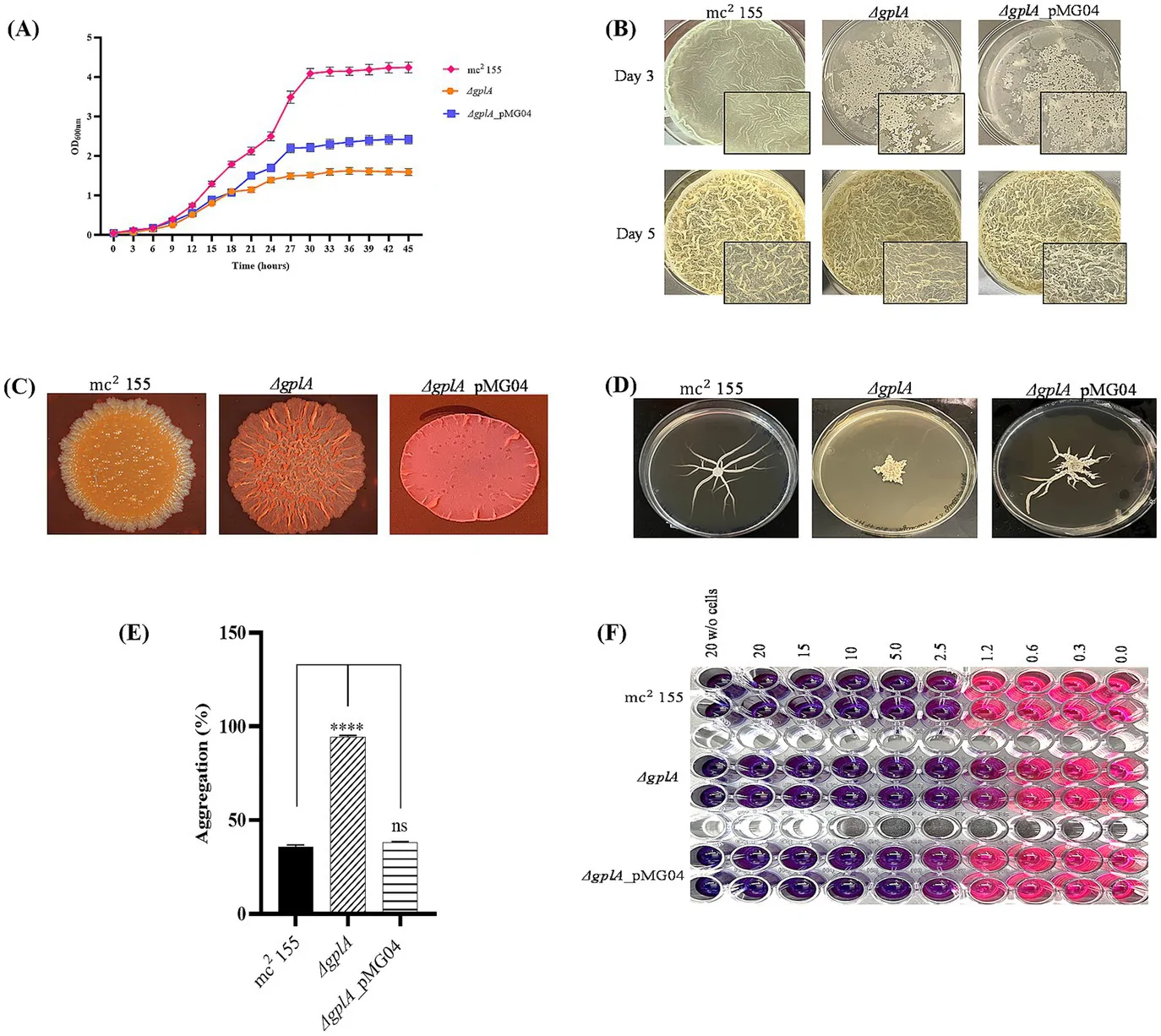
Deletion of *gplA* alters the physiology of *M. smegmatis*. Phenotypic comparison of wild-type *M. smegmatis* (mc^2^155), the *MSMEG_0394* null mutant (*ΔgplA*), and the complemented strain (*ΔgplA_*pMG04). **(A)** Planktonic growth under standard growth conditions, obtained by plotting OD_600_ of the liquid culture at different time points. Data represent the mean of at least three independent experiments, and error bars indicate standard deviation. **(B)** Biofilm formation at the air-liquid interface after 3 and 5 days of incubation. **(C)** Colony morphology on Congo red agar plate assay. **(D)** Sliding motility on semi-solid medium. **(E)** Cell aggregation percentage. Error bars are representative of the SD of three independent experiments. The significance of data obtained was tested by the ordinary one-way ANOVA with Sidak’s multiple comparisons (**** *p* < 0.0001; ns, Not significant). **(F)** Minimum inhibitory concentration (MIC) of rifampicin for mc^2^155 (2.5 μg mL^−1^), Δgp*lA* (1.2 μg mL^−1^), and *ΔgplA_*pMG04 (2.5 μg mL^−1^). For panels **(B–D)**, **(F)** experiments were performed in triplicate, and representative images are shown.

Many phenotypes, such as growth rate, colony morphology, sliding motility, biofilm formation, autoaggregation, and antibiotic susceptibility, are well established to be influenced by GPLs biosynthesis and their surface exposure in *Mycobacterium* spp. (Johansen et al., 2020a; Kocíncová et al., 2008; Recht and Kolter, 2001; Ryan and Byrd, 2018; Sondén et al., 2005). Alterations in surface-exposed GPLs profoundly affect the physicochemical properties of the mycobacterial cell envelope, thereby affecting both physiology and drug response (Deshayes et al., 2005; Etienne et al., 2002; Tatham et al., 2012). In the present study, a *M. smegmatis* mutant strain carrying a deletion of the 228 bp *MSMEG_0394* gene was generated, *ΔMSMEG*_*0394* (hereafter referred to as *ΔgplA*). Deletion of *gplA* did not affect the transcription of the remaining operon *MSMEG_0394/95*, as demonstrated by RT-PCR analysis described above (Figure 2A). In order to elucidate the functional role of GplA, predicted to be involved in GPL homeostasis, we investigated whether deletion of the coding gene, impacts the key surface-associated phenotypes in *M. smegmatis*. Representative results for these phenotypes are shown in Figure 3. A first notable effect was observed in growth dynamics (Figure 3A). The estimated generation times during exponential growth were similar among the three strains (approximately 3.5 h, 3.0 h, and 3.7 h for the wild-type, *ΔgplA* mutant, and complemented strain, respectively), indicating that deletion of *gplA* did not markedly affect exponential growth. However, clear differences emerged during the transition to stationary phase. While wild-type (wt) cultures reached an OD_600_ of ~4.2, the *ΔgplA* mutant plateaued earlier at ~1.6. Complementation partially restored growth, reaching intermediate levels. This impaired growth is consistent with the multifaceted role of GPLs in maintaining envelope stability and facilitating nutrient exchange, suggesting that the absence of *gplA* imposes a physiological burden on the cells, reducing their fitness *in vitro*. The deletion also resulted in a striking alteration of colony morphotype (Figure 3C). When cultivated on Congo red agar under carbon-limiting conditions, *M. smegmatis* wild-type developed smooth, reddish colonies with a glossy, translucent appearance, consistent with previous reports (Sondén et al., 2005; Tatham et al., 2012). In contrast, the Δ*gplA* mutant displayed a markedly altered phenotype, forming rough and opaque colonies with a dried appearance. Complementation did not fully restore the wild-type morphology, as the complemented strain formed colonies different from those of the mutant, but still retaining a rough and opaque appearance. Sliding motility was also compromised in *ΔgplA* strain (Figure 3D). While wild-type cells exhibited active surface translocation, producing finger-like extensions from the inoculation point, consistent with previous reports (Etienne et al., 2002; Pollitt et al., 2025), the *ΔgplA* strain showed no detectable motility. The complemented strain regained this ability, indicating complete functional restoration. These findings are consistent with the established role of GPLs in reducing surface tension and enabling lateral expansion on semisolid substrates (Etienne et al., 2002). Thus, the motility defect observed in the Δ*gplA* mutant is consistent with the altered GPL profile and supports the hypothesis that GplA contributes to GPL homeostasis required for efficient surface translocation. This alteration may also explain the mutant’s reduced ability to colonize interfaces during the initial stages of biofilm formation. Biofilm formation assays further supported this view (Figure 3B). Wild-type cells produced uniform pellicles along the air-medium interface by day 3 of incubation, which matured by day 5. In contrast, the *ΔgplA* strain initially formed suspended aggregates and sparse surface foci, although it eventually produced a textured pellicle by day 5. Surprisingly, the complement strain shows the same kinetics as the mutant strain. These observations suggest that GPLs facilitate surface colonization and lateral spreading, likely by reducing cell–cell cohesion and enabling surface motility (Yang et al., 2017). Consistent with this hypothesis, the *ΔgplA* mutant showed significantly higher autoaggregation (~95%) compared to the wild-type (~35%), reflecting enhanced cell–cell interactions likely contribute to the increased stability and density of the mutant biofilms. This phenotype was reversed by complementation, which restored the aggregation index to wild-type levels (Figure 3E). Despite its pronounced effects on colony morphology, biofilm formation, aggregation, motility, and growth, *gplA* deletion had only a modest impact on antimicrobial susceptibility. Antibiotic susceptibility testing using the Resazurin Reduction Microtiter Assay (REMA) showed an increased sensitivity of the *ΔgplA* strain to rifampicin (MIC: 1.2 μg mL^−1^) compared with wild-type (MIC: 2.5 μg mL^−1^) (Figure 3F), while susceptibility to streptomycin, ethambutol, ciprofloxacin, norfloxacin, and chloramphenicol remained unchanged (Supplementary Figure S2). Complementation restored rifampicin resistance to wt levels. The increased susceptibility to rifampicin may reflect subtle alterations in membrane composition or fluidity resulting from altered GPL homeostasis, consistent with the role of the outer membrane as a permeability barrier (Tursun et al., 2025). However, the modest change in rifampicin MIC, together with the lack of effect on other antibiotics, suggests that although *gplA* appears to be involved in GPL homeostasis, its contribution to the cell wall component responsible for drug permeability is not decisive. Collectively, these results demonstrate that *gplA* plays an essential role in maintaining surface-associated phenotypes in *M. smegmatis*, including colony morphology, biofilm formation, cell–cell interactions, motility, and overall fitness.

### 3.4 Deletion of *gplA* affects the GPLs profile

**Figure 4:**
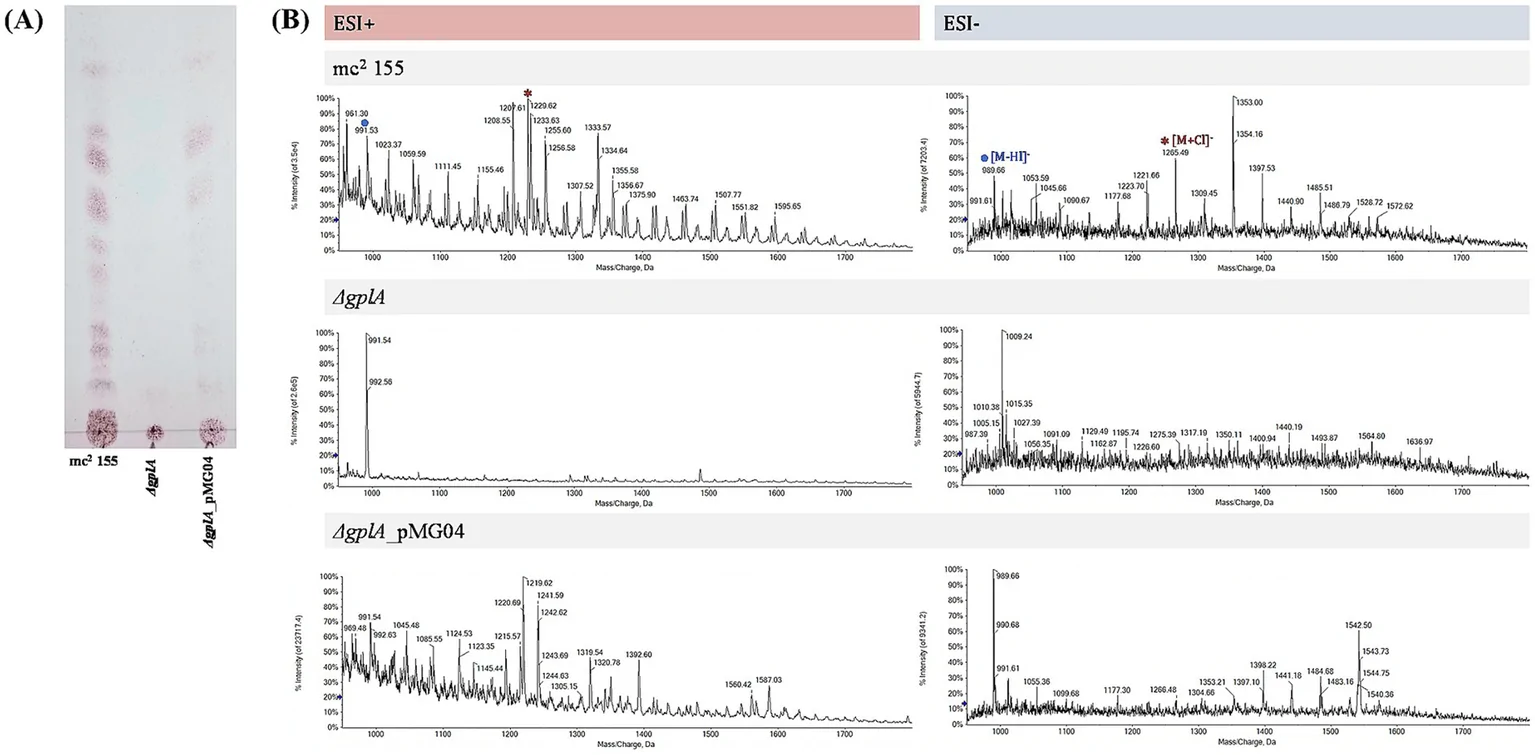
Total lipid extracts analysis. **(A)** Thin-layer chromatography (TLC) analysis of total lipid extracts from *M. smegmatis* mc2155 (wild-type), *ΔgplA*, and the complemented strain *ΔgplA*_pMG04. **(B)** Full-scan ESI-MS analysis of GPL extracts acquired in positive-ion (ESI+) and negative-ion (ESI-) modes.

GPL production was evaluated using a combined chromatographic and mass-spectrometric approach to compare strain-dependent differences in the GPL profile. Total lipid extracts were first analyzed by thin-layer chromatography (TLC) (Figure 4A), which was used as a qualitative comparative technique to assess differences in GPL-associated lipid profiles among the wild-type, *ΔgplA* mutant, and complemented strains. Due to the qualitative nature of TLC and the absence of purified GPL standards, this analysis was intended to compare migration patterns rather than to identify individual GPL molecular species. The TLC profiles revealed marked qualitative differences among the three strains (Etienne et al., 2005). The wild-type strain exhibited a characteristic pattern of multiple GPL-associated bands distributed across both apolar and polar regions of the plate, consistent with the presence of a chemically diverse and structurally heterogeneous GPL population. In contrast, the *ΔgplA* mutant displayed a substantial reduction in both the number and intensity of GPL-associated bands, indicating a pronounced alteration in GPL biosynthesis. The complemented strain showed partial restoration of the wild-type profile, with reappearance of several GPL-associated bands, albeit at reduced intensity, indicating substantial recovery of the native GPL repertoire. To further investigate these differences at the molecular level, GPL extracts were subsequently analyzed by electrospray ionization mass spectrometry (ESI-MS) using full-scan acquisition in both positive- and negative-ion modes (Figure 4B). In both ionization polarities, GPL-associated ions accounted for the majority of the detected lipid signals, indicating that GPLs constitute the dominant lipid class in the analyzed extracts (Frankfater et al., 2020). This close correspondence between the TLC profiles and the full-scan MS data supports the conclusion that the observed strain-dependent differences reflect changes in GPL composition. The inspection of the spectra shows that positive-ion mode yields a higher signal-to-noise ratio, a denser distribution of GPL-associated peaks, and clearer strain-specific differences, whereas negative-ion mode provides less intense but fully consistent qualitative patterns (Hsu et al., 2012). In positive-ion mode, the wild-type strain displayed a highly complex mass spectrum characterized by numerous GPL-associated ions distributed over a broad *m/z* range (approximately 1,050–1700), indicative of an extensive population of structurally related GPL species. The complemented strain exhibited a similar overall distribution, although with reduced spectral density and signal intensity, consistent with partial restoration of GPL diversity. In contrast, the *ΔgplA* mutant showed a markedly simplified spectrum in which most higher *m/z* GPL-associated ions were absent, and the signal was dominated by a small number of lower-mass species. Within this simplified profile, a prominent ion at *m/z* 991 (positive-ion mode) was reproducibly detected in all three strains, including the *ΔgplA* mutant, where it represented one of the dominant features of the spectrum (Frankfater et al., 2020). In negative-ion mode, a closely related ion at *m/z* 989 was clearly detected in the wild-type and complemented strains but was consistently absent in the *ΔgplA* mutant. Given that this ion is readily detectable under the same acquisition conditions in the wild-type and complemented strains, its absence in the deletion mutant may reflect reduced abundance below detection limits rather than true absence. The persistence and relative dominance of the ion detected at a nominal *m/z* of 991 in the deletion background are consistent with a core GPL-associated molecular form. The concurrent reduction of higher-mass GPL-associated ions in the *ΔgplA* mutant further suggests that GplA is involved in downstream modification or maturation of pre-existing GPL species rather than in formation of the core GPL scaffold. Because these analyses were based on full-scan ESI-MS in both ionization polarities, the observed differences were interpreted in terms of global changes in GPL population structure and maturation states, rather than definitive structural assignments of individual molecular species. Nonetheless, the coordinated loss of higher-m/z GPL-associated ions in the *ΔgplA* mutant and their recovery following complementation strongly indicate that GplA contributes to the generation of the mature GPL repertoire. The persistence of the GPL-associated ion at m/z 991 across all strains suggests that the core GPL scaffold can still be produced in the absence of *gplA*, whereas the reduced representation of higher-mass species points to a role in downstream maturation or modification steps. Given that GPL structural diversity in mycobacteria is generated through tailoring reactions such as methylation, acetylation, and glycosylation (Schorey and Sweet, 2008), the simplified GPL profile of the *ΔgplA* mutant is consistent with impaired processing of GPL molecules rather than a complete block in GPL biosynthesis.

### 3.5 Structural and dynamical characterization of GplA and the homologous MAB-4102c protein

**Figure 5:**
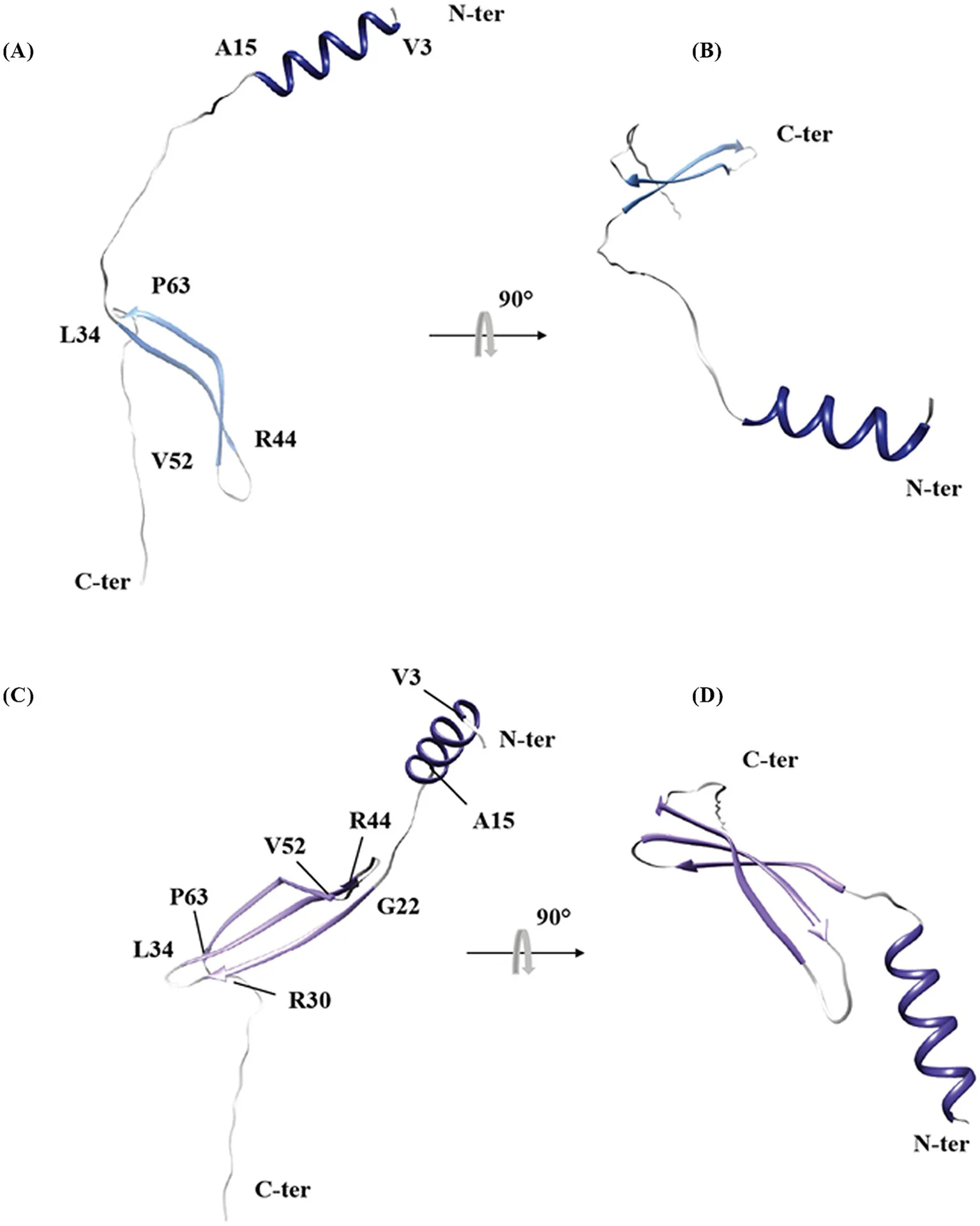
Ribbon drawing representation of the 3D structural models of GplA **(A,B)** and MAB-4102c **(C,D)** proteins obtained using the AlphaFold approach. The residues located at the N- and C-end of each secondary structure element are highlighted. The secondary structure elements were identified, as described in the materials and methods section, using DSSP software.

**Figure 6:**
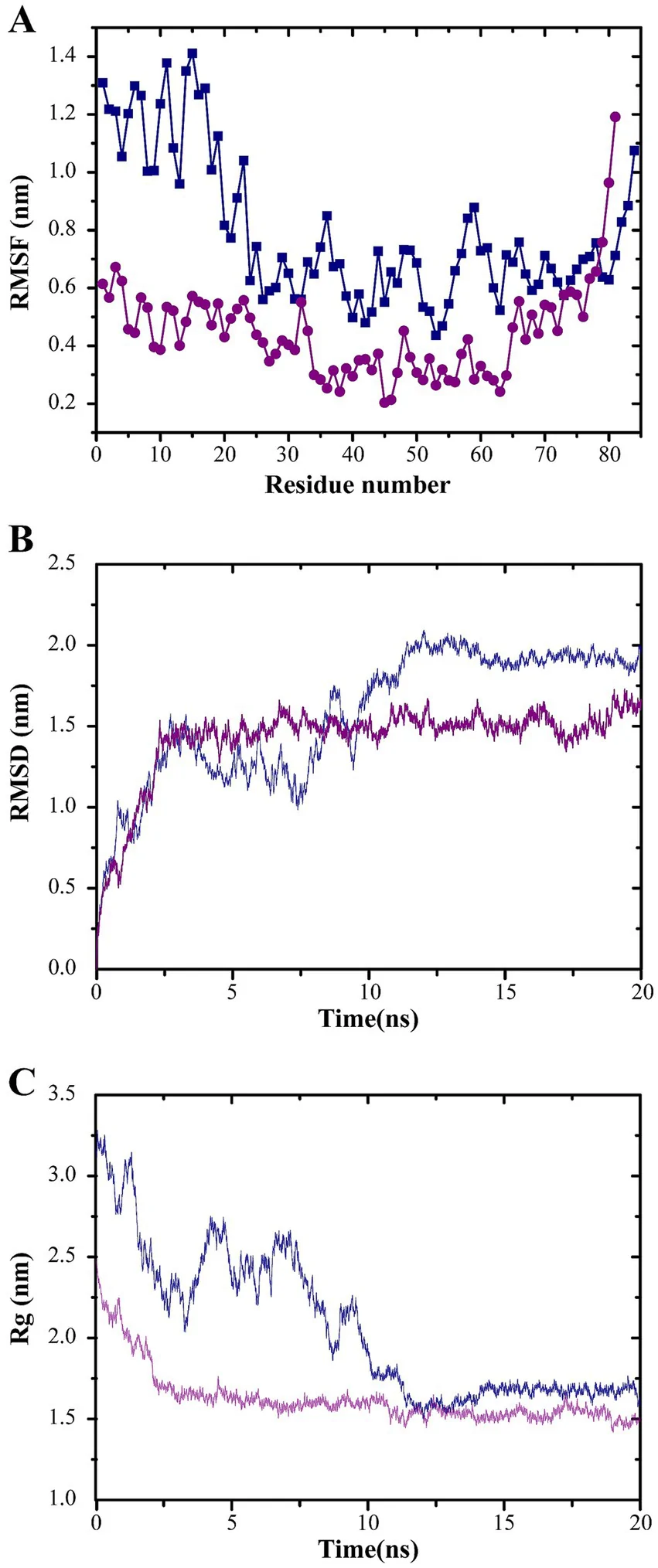
Molecular dynamics simulation results obtained for GplA (blue) and MAB-4102c (purple). Per-residue root mean square fluctuation (RMSF) (nm) **(A)**, root mean square deviation (RMSD) (nm) **(B)** and radius of gyration (Rg) (nm) **(C)** as a function of time.

To gain structural insights into GplA and assess whether its structural features are conserved in clinically relevant non-tuberculous mycobacteria, we compared GplA with its *M. abscessus* homologue MAB-4102c. Homology modelling followed by molecular dynamics (MD) simulations was employed to characterize the structural organization and conformational dynamics of both proteins. The 3D structural models predicted using the AlphaFold (Abramson et al., 2024) approach indicate that the two proteins are characterized by a similar elongated structural organization with secondary structure elements linked by long flexible unstructured regions (Figures 5A–D). In the case of GplA, the tertiary structure is composed of a one N-terminal *α*-helix encompassing residues Val3-Ala15 and two *β*-strands (*β*1 = Leu34-Arg44; *β*2 = Val52-Pro63) (Figures 5A, B); whereas MAB-4102c presents a three-dimensional architecture in which the N-terminal α-helix, containing the residues from Val3-Ala15, is linked by a long flexible region to three *β*-strands (*β*1 = Gly22-Arg30; *β*2 = Leu34-Arg44; *β*3 = Val52-Pro63) (Figures 5C,D). After that, in order to understand the relationship between the molecular motions of GplA and MAB-4102c and their structural and functional properties, we investigated the dynamic personalities of both proteins by MD simulation methods that are able to provide a detailed description of proteins and nucleic acids’ dynamical process at the picoseconds to milliseconds timescale (Lindorff-Larsen et al., 2005; Lindorff-Larsen et al., 2011; Anand et al., 2022; Kumar et al., 2021). First, we generated for each protein, as described in the materials and methods section, a conformational ensemble reproducing backbone dynamics on the picosecond to nanosecond timescale. Then, we investigated the backbone conformational mobility by analyzing the per-residue root mean square fluctuation (RMSF) values from each of the conformational ensembles obtained for GplA and MAB-4102c, respectively (Figure 6A). The analysis of the RMSF values indicates that in both proteins, the largest deviation occurs at the N-terminal regions (Figure 6A). Yet, the analysis of the Root mean square deviation (RMSD) (Figure 6B) and of the radius of gyration (Rg) (Figure 6C) (for GplA and MAB-4102c indicates that both proteins reached equilibrium during the 20 ns trajectory of MD simulation.

### 3.6 Electrostatic surface analysis of GplA and MAB-4102c

**Figure 7:**
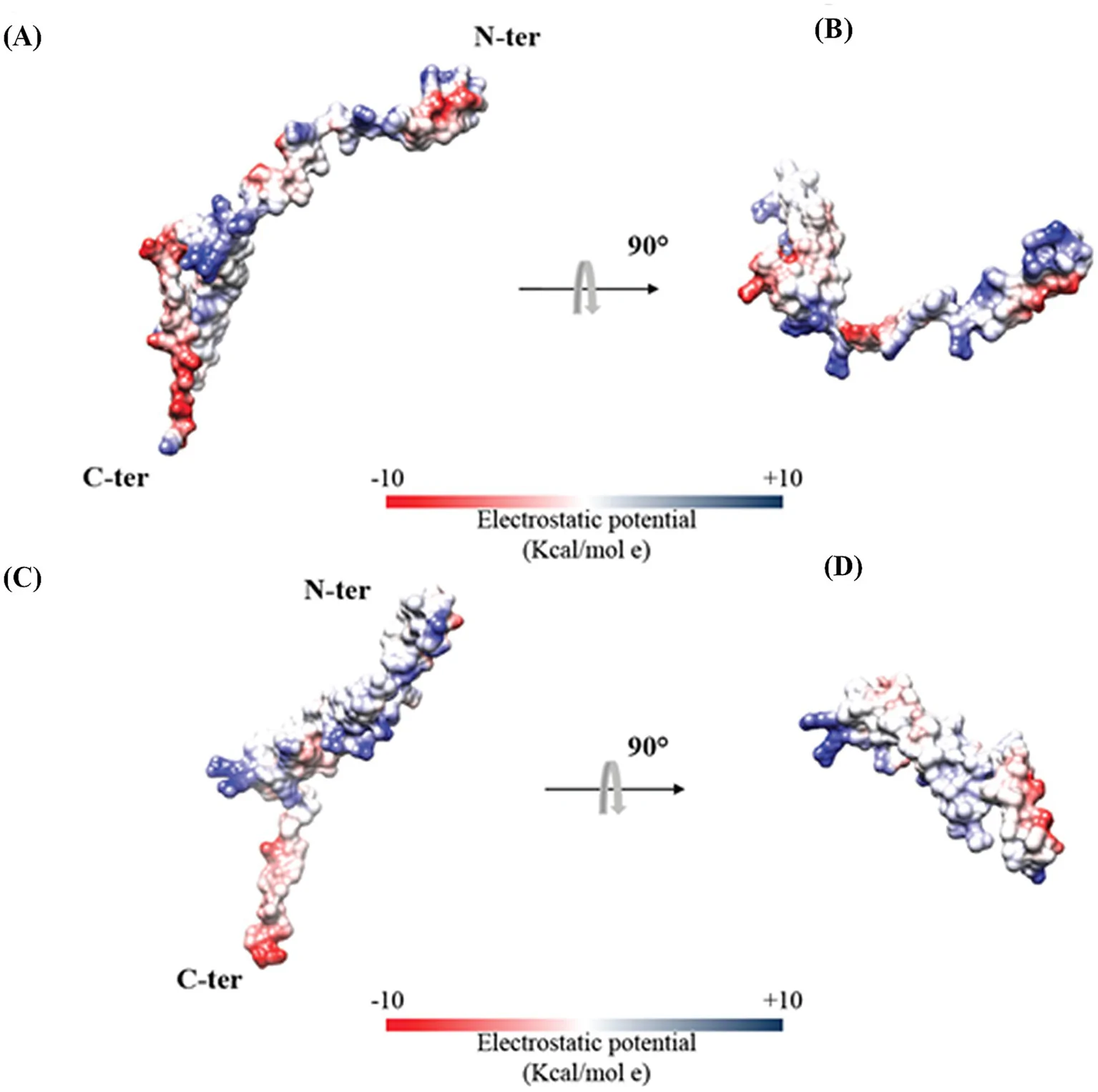
Surface electrostatic potential of GplA and MAB-4102c. Coulombic electrostatic potential mapped onto the surface of GplA **(A,B)** and MAB-4102c **(C,D)** in two different orientations. The surface of each protein is depicted from electropositive (blue; 10 kcal/mol) to electronegative (red; −10 kcal/mol).

Using the UCSF Chimera bioinformatic tool, the Coulombic electrostatic potential of the predicted structural models of GplA and MAB-4102c was calculated (Figure 7). To note, the two proteins are characterized by a similar but not identical electrostatic surface. The electrostatic potential map of a protein may suggest a putative function for it. The electrostatic distribution of GplA and MAB-4102c, due to the poorly represented positive charges, may exclude a principal role of the two proteins as DNA binder (blue portion; Figure 7). Moreover, the high content of exposed charged amino acid residues and polar amino acids, which makes both proteins highly soluble, supports the hypothesis of a role for GplA and MAB-4102c in protein–protein interactions in the cytosol rather than in membrane localization.

### 3.7 GplA regulates the transcription of *MSMEG_0393/94/95* operon

**Figure 8:**
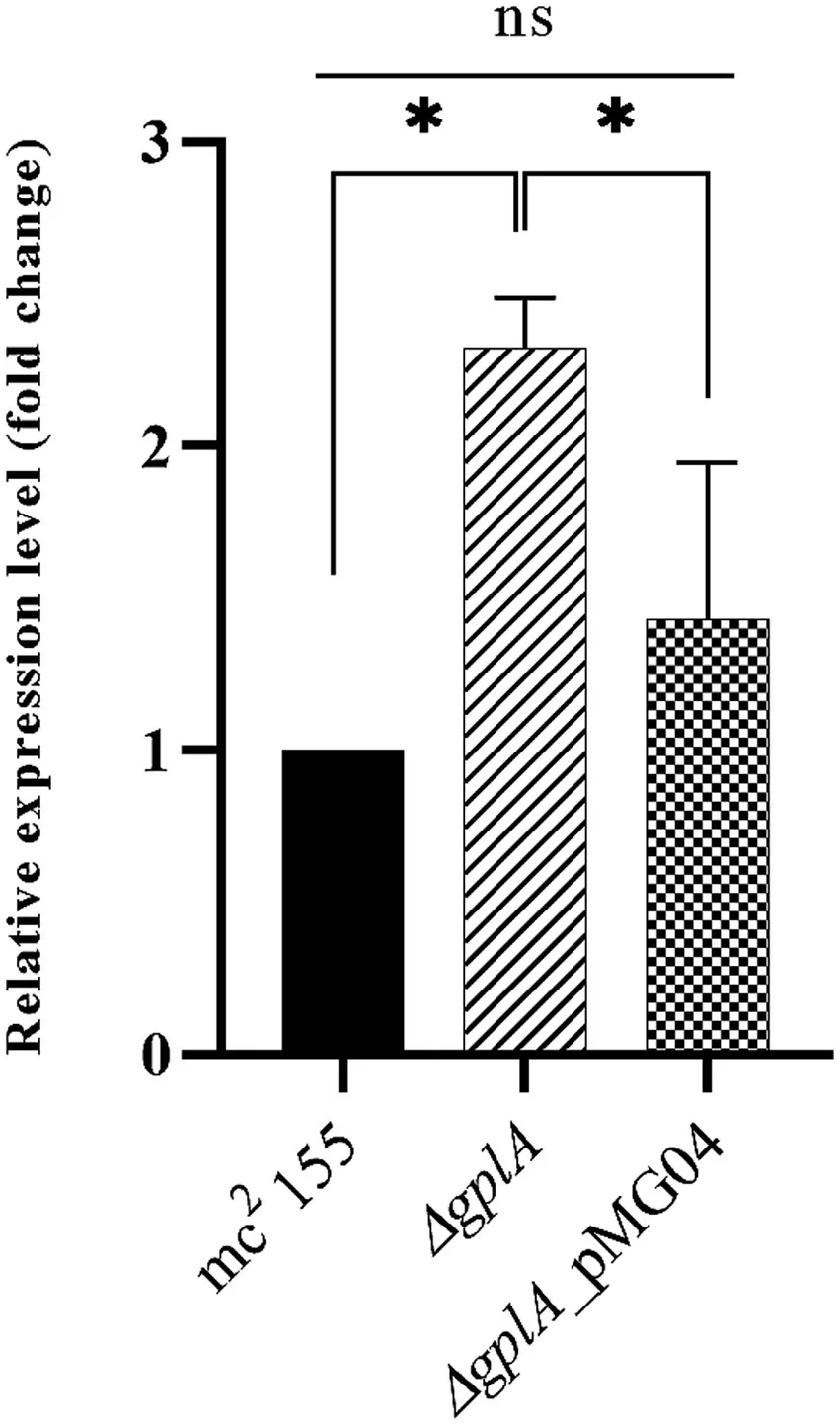
RT-qPCR analysis of the *MSMEG_0393/95* operon. Comparative analysis of the expression of the transcriptional unit was carried out on total RNAs extracted from *M. smegmatis* mc2155, *ΔgplA,* and complemented *ΔgplA_*pMG04 strains. As a reference gene, *sigA* was used. Relative expression levels were calculated with the $2^{-\text{ΔΔCt}}$ method; data shown are the mean values of two independent experiments, and each RT-qPCR was carried out in technical triplicate. Error bars represent the standard deviation of the mean values. The significance of data obtained was tested by the Ordinary One-way ANOVA with Sidak’s multiple comparisons (*p < 0.05; ns, Not significant).

Bioinformatic analysis based on the primary sequence and the surface electrostatic distribution suggests a possible role of GplA in transcriptional regulation, working through protein–protein interactions. In order to corroborate this hypothesis, qRT-PCR analysis was performed to investigate the effect of the deletion of *gplA* on the expression of its own operon. The qRT-PCR experiments revealed that deletion of *MSMEG_0394* led to a 2.5-fold upregulation of *MSMEG_0393/95* transcription, supporting the role of GplA as a negative regulator of its own operon. Reintroduction of *gplA* under the constitutive *hsp60* promoter restored repression, returning expression almost to wild-type levels (Figure 8).

## 4 Discussion

Glycopeptidolipids are major determinants of cell-envelope architecture and surface-associated physiology in NTM, influencing colony morphology, biofilm formation, motility, antibiotic susceptibility and host interactions (Gutiérrez et al., 2018; Tursun et al., 2025). Although the biosynthetic pathway of GPLs has been extensively investigated in *M. smegmatis*, the function of several conserved genes within the GPL locus remains unresolved (Hsu et al., 2012; Johansen et al., 2020b). In the present study, we functionally characterized GplA (MSMEG-0394) and identify it as a previously unrecognized determinant of GPL homeostasis. By combining genetic, biochemical, transcriptional and structural analyses, we demonstrate that deletion of *gplA* is associated with coordinated alterations in GPL composition, multiple surface-associated phenotypes and operon expression, supporting a role for GplA in maintaining cell-envelope homeostasis. Transcriptional analysis demonstrated that *gplA* is the second gene of a three-gene operon including *MSMEG_0393*, encoding a predicted fatty-acid O-methyltransferase, and *MSMEG_0395*, encoding a hypothetical protein. Deletion of *gplA* profoundly affected colony morphology, sliding motility, biofilm formation, autoaggregation and growth kinetics. Taken together, these phenotypes indicate a coordinated alteration of cell-envelope-associated functions rather than independent physiological defects. Similar phenotypes have consistently been reported in mutants defective in established GPL biosynthetic enzymes, including glycosyltransferases and methyltransferases (Deshayes et al., 2005; Etienne et al., 2002; Illouz et al., 2023). In particular, the rough colony morphology, increased aggregation, and impaired motility observed in the *ΔgplA* strain are consistent with alteration in surface-exposed GPLs, which normally modulate cell-surface hydrophobicity and reduce surface tension (Etienne et al., 2002; Tatham et al., 2012). The partial restoration of these traits in the complemented strain further supports the involvement of GplA in maintaining GPL-dependent envelope properties.

Thin-layer chromatography (TLC) and electrospray ionization-mass spectrometry (ESI-MS) corroborated the phenotypic observations, revealing marked alterations in the GPL profile of the *ΔgplA* mutant. In particular, the mutant displayed a simplified GPL-related mass profile characterized by a reduction in higher *m/z* species and a redistribution of signal intensity toward lower-mass GPL-associated ions, indicating remodeling of the GPL repertoire. Rather than indicating a complete loss of GPL biosynthesis, the combined TLC and ESI-MS analyses support a role for GplA in maintaining GPL homeostasis, possibly by preserving the correct balance of GPL maturation or modification.

Deletion of *gplA* also resulted in upregulation of the *MSMEG_0393/0395* operon. Because *MSMEG_0393* encodes a predicted O-methyltransferase, increased expression of this gene may contribute to alterations in methylation-dependent GPL modifications. Similar relationships between methyltransferase activity and GPL modification have previously been reported in *M. smegmatis* and *M. abscessus* (Daher et al., 2020; Jeevarajah et al., 2002). Collectively, these findings suggest that GplA contributes, directly or indirectly, to maintaining appropriate expression of the *MSMEG_0393/94/95* operon. Although the underlying molecular mechanism remains unknown, the transcriptional response observed in the mutant is consistent with a role for GplA in maintaining GPL homeostasis rather than in direct transcriptional regulation.

Structural modelling indicates that GplA is a small soluble protein lacking recognizable catalytic features, making a direct enzymatic role in GPL biosynthesis unlikely. Instead, its predicted structural properties are consistent with an indirect function, potentially mediated by protein–protein interactions. Although this hypothesis remains to be experimentally validated, small proteins acting through protein–protein interactions have been implicated in the regulation of numerous bacterial processes, including morphogenesis, cell division, enzymatic activity and stress responses (Quendera et al., 2020; Ul Haq et al., 2020). Thus, GplA may represent a previously unrecognized regulatory component acting upstream of GPL-associated pathways rather than a catalytic enzyme.

Interestingly, complementation of *ΔgplA* under the constitutive *hsp60* promoter restored several, but not all, phenotypes to wild-type levels. While autoaggregation, sliding motility, MIC of rifampicin, and operon expression are fully restored in the complemented strain, the growth phenotype, colony morphology, biofilm development, and GPL profile were only partially rescued. Such incomplete complementation is not uncommon in mycobacteria, since heterologous constitutive expression frequently fails to reproduce the native temporal or quantitative regulation required for full phenotypic recovery (Lefebvre et al., 2018; Tatham et al., 2012; Tsai et al., 2013). These observations further suggest that appropriate temporal and quantitative expression of GplA is required to maintain physiological GPL homeostasis.

## 5 Conclusion

Collectively, our findings identify GplA as a previously unrecognized factor associated with GPL homeostasis in *M. smegmatis*. Its absence leads to coordinated alterations in GPL composition, surface-associated phenotypes and its own operon expression, providing new insight into the regulatory mechanisms that contribute to cell-envelope integrity. Given the conservation of GplA homologues among clinically relevant NTM, including *M. abscessus* and *M. chelonae* (Johansen et al., 2020b; Ripoll et al., 2007), elucidating its molecular partners and mechanism of action will be an important focus of future studies. A deeper understanding of these regulatory pathways will advance our knowledge of GPL homeostasis and cell-envelope adaptation in NTM.

## Data Availability

All datasets generated and analyzed during this study are included in this published article, its Supplementary material, or in the Zenodo repository (https://doi.org/10.5281/zenodo.18174085).

## Glossary

GPLs: glycopeptidolipids
NTM: non-tuberculous mycobacteria
MAC: *Mycobacterium avium* complex
PIM2: proviral integrations of moloney virus 2
TLR-2: toll-like receptors-2
TNF: tumor necrosis factor
TLC: thin-layer chromatography
ESI-MS: direct infusion electrospray ionization-mass spectrometry
LB: luria-bertani
MB7H9: Middlebrook 7H9
ADC: albumin-dextrose-catalase
X-gal: 5-bromo-4-chloro-3-indolyl-*β*-d-galactopyranoside
MSA: multiple sequence alignment
pLDDT: predicted local distance difference test
TIP3P: transferable intermolecular potential with 3 points
PCR: polymerase chain reaction
SCO: single-crossover
RT-PCR: reverse transcription polymerase chain reaction
RTq-PCR: reverse transcription quantitative polymerase chain reaction
WT: wild-type
PBS: phosphate-buffered saline
MIC: minimum inhibitory concentration
REMA: Resazurin Reduction Microtiter Assay
TSB: tryptic soy broth
RT: room temperature
IS: ion spray
CUR: curtain gas.
GS1: nebulizer gas
GS2: auxiliary gas
TEM: source temperature
DP: declustering potential
EP: entrance potential
PAN: protein accession numbers
GLT: genomic locus tags
GA: genome annotation
MD: molecular dynamics
RMSF: root mean square fluctuation
