# Supplementary material for: Functional characterization of GplA (MSMEG-0394) reveals a novel determinant of glycopeptidolipids homeostasis in Mycobacterium smegmatis
Source: Front Microbiol. 2026 Sep 3;17:1929615. doi: 10.3389/fmicb.2026.1929615 (PMC13582512; doi:10.3389/fmicb.2026.1929615)
Supplement: Supplementary file 1 [file Supplementary_file_1.DOCX]

**Functional characterization of GplA (MSMEG_0394) reveals a novel determinant of glycopeptidolipids homeostasis in *Mycobacterium smegmatis***

Milena Della Gala^1^, Rosangela Marasco^1^, Ida De Chiara^1^, Martina Montebuglio^2^, Luigi Russo^1^, Severina Pacifico^1^, Lidia Muscariello^1^*

^1^Department of Environmental, Biological and Pharmaceutical Sciences and Technologies (DiSTABiF), University of Campania Luigi Vanvitelli, Caserta, Italy

^2^Institute of Biostructure and Bioimaging (IBB), National Research Council (CNR), Napoli, Italy

***Supplementary Material***

**Supplementary Figure S1.** Transcriptional analysis of the *MSMEG_0394* locus. RT-PCR experiments were performed on total RNAs extracted from wild-type (*MSMEG_0393/94/95*) and mutant (*MSMEG_0393/95*) strains. Each set of three reactions contains a positive control with genomic DNA as the template (C), a negative control without reverse transcriptase (-), and total RNAs with reverse transcription step (+). MW, molecular weight marker (1kb Plus Opti-DNA Marker, abm). The red box indicates the region of the gel that was cropped and presented in the figure included in the main text (Figure 2A)

**Supplementary Figure S2.** Minimum inhibitory concentration (MIC) analyses by the Resazurin Reduction Microtiter Assay (REMA) for (A) ciprofloxacin, (B) streptomycin, (C) norfloxacin, (D) ethambutol, (E) isoniazid, and (F) chloramphenicol, performed on the mc^2^155, *ΔgplA*, and *ΔgplA*_pMG04 *M. smegmatis* strains.
